# Non-enzymatic role of SOD1 in intestinal stem cell growth

**DOI:** 10.1038/s41419-022-05267-w

**Published:** 2022-10-20

**Authors:** Ying-Chao Wang, Xiao-Xu Leng, Cheng-Bei Zhou, Shi-Yuan Lu, Chi Kwan Tsang, Jie Xu, Ming-Ming Zhang, Hui-Min Chen, Jing-Yuan Fang

**Affiliations:** 1 Division of Gastroenterology and Hepatology; Shanghai Institute of Digestive Disease; NHC Key Laboratory of Digestive Diseases; State Key Laboratory for Oncogenes and Related Genes; Renji Hospital, School of Medicine, Shanghai Jiao Tong University, Shanghai, China; 2 Clinical Neuroscience Institute, The First Affiliated Hospital of Jinan University, Guangzhou, China; 3 Institutes of Biomedical Sciences, Zhongshan-Xuhui Hospital, Fudan University, Shanghai, China

**Keywords:** Intestinal stem cells, Cancer prevention, Extracellular signalling molecules, Growth factor signalling, Apoptosis

## Abstract

Superoxide dismutase 1 (SOD1) modulates intestinal barrier integrity and intestinal homeostasis as an antioxidant enzyme. Intestinal homeostasis is maintained by the intestinal stem cells (ISCs). However, whether and how SOD1 regulates ISCs is unknown. In this study, we established intestinal organoids from tamoxifen–inducible intestinal epithelial cell–specific *Sod1* knockout (*Sod1*^*f/f*^*; Vil-creERT2*) mice. We found that loss of *Sod1* in organoids suppressed the proliferation and survival of cells and *Lgr5* gene expression. SOD1 is known for nearly half a century for its canonical role as an antioxidant enzyme. We identified its enzyme-independent function in ISC: inhibition of SOD1 enzymatic activity had no impact on organoid growth, and enzymatically inactive *Sod1* mutants could completely rescue the growth defects of *Sod1* deficient organoids, suggesting that SOD1-mediated ISC growth is independent of its enzymatic activity. Moreover, *Sod1* deficiency did not affect the ROS levels of the organoid, but induced the elevated WNT signaling and excessive Paneth cell differentiation, which mediates the occurrence of growth defects in *Sod1* deficient organoids. In vivo, epithelial *Sod1* loss induced a higher incidence of apoptosis in the stem cell regions and increased Paneth cell numbers, accompanied by enhanced expression of EGFR ligand Epiregulin (EREG) in the stromal tissue, which may compensate for *Sod1* loss and maintain intestinal structure in vivo. Totally, our results show a novel enzyme-independent function of SOD1 in ISC growth under homeostasis.

## Introduction

Intestinal stem cell (ISC) populations are crucial for intestinal homeostasis by undergoing either self-renewing or differentiation divisions. Cells in different locations in the crypt can act as ISCs in the mouse intestine.

Crypt base columnar cells (CBCs) which are named because of their elongated/columnar appearance, reside at the bottom of the crypts, intermingled with the Paneth cells. CBCs are rapidly cycling and show characteristic expression of *Lgr5* [1]) and *Olfm4* [2]. The CBCs form rapidly proliferating transit-amplifying (TA) progenitors that differentiate downwards into Paneth cells at the crypt base, and upwards along the crypt-villus axis into enterocytes, goblet cells and enteroendocrine cells, which undergo apoptosis at the tips of the villi. Under normal conditions, continuous exposure to food and bacteria leads to enhanced apoptosis and removal of mucosal cells. The CBCs are responsible for replenishment of lost epithelial cell populations [1]. Another stem cell pool is present above the Paneth cells at position +4 relative to the base of the crypt. However, there is no consensus regarding their cycling dynamics and sensitivity to irradiation. Numerous reports have showed that +4 ISCs are slow-cycling, radioresistant, and are marked by the expression of *Bmi1*, *mTert*, and *Hopx* [3–5]. Under homeostatic conditions, quiescent *Bmi1*^+^ ISCs are slow-cycling. Upon intestinal injury or ablation of *Lgr5*, the quiescent *Bmi1* or *Hopx*-expressing cells can enter a rapidly cycling state to replenish the *Lgr5*^+^ ISCs as the reserve stem cell pool [5, 6]. Potten et al., however, proposed that the position +4 harbors fast-cycling and radiosensitive ISCs based on lineage-tracing data. A dose of irradiation as low as 1 Gy is sufficient to eliminate all Potten’s +4 cells [7, 8] comprising both *Lgr5*-positive and *Lgr5*-negative cells [1, 9]. The radiosensitivity of +4 populations is a protective mechanism preventing the transmission of irradiation-induced damaged DNA to progenitors [10]. The radiosensitive +4 cells are distinct from active CBCs or quiescent +4 populations which have a low sensitivity to irradiation [3, 11, 12]. Once the radiosensitive +4 cells are eliminated, the neighboring proliferative progenitors, including CBCs, will efficiently replace them and assume their functions [9, 13].

Paneth cells are the only differentiated cells at the bottom of the crypt and protect the intestinal barrier by secreting antimicrobial granules containing defensin, cryptdins and lysozyme (LYZ) [14]. Besides the antimicrobial function, Paneth cells act as a critical component of the ISCs niche by secreting essential signals such as EGF, R-spondin1 (RSPO1), and WNT3a [15, 16]. Paneth cell-derived WNT signaling not only supports ISC growth [17, 18] but also functions as a differentiation signal for Paneth cells and drives Paneth cell maturation and differentiation. In the absence of WNT signals, Paneth cells exhibit improper localization and fail to undergo morphological maturation [19]. Upon activation of WNT signaling, β-catenin/TCF4 complex activates the transcription of Paneth cells markers and promotes ISCs differentiation towards the Paneth cell lineage [19, 20]. The ISCs growth and differentiation should be tightly regulated to keep homeostasis. Although multiple signaling pathways were involved in ISCs regulation [21], their exact mechanisms remain unclear.

The Superoxide dismutase (SOD) family of antioxidant enzymes consist of SOD1 (Cu/Zn superoxide dismutase, Cu/Zn-SOD), SOD2 (manganese superoxide dismutase, Mn-SOD), and SOD3 (extracellular superoxide dismutase, EC-SOD). SOD1 is distributed in the cytosol, nucleus, peroxisomes, and mitochondrial intermembrane space [22]. Its canonical function is as antioxidant enzymes sustaining redox balance by catalyzing the rapid conversion of the superoxide anion into molecular oxygen and hydrogen peroxide [23]. On the other hand, SOD1 also exerts an enzyme-independent function, such as the regulation of the genes expression as a transcriptional factor [24–27], and the regulation of ribosome biogenesis driving KRAS-driven lung tumorigenesis [28]. It is known that SOD1 is closely associated with epithelial barrier integrity. *Sod1* deficiency or mutation causes redox imbalance which leads to disruption of intestinal tight junction in vivo [29, 30]. *Sod1* overexpression significantly reduces intestinal damage induced by intestinal ischemia/reperfusion [31–33]. Given the critical role of ISCs in keeping the intestinal barrier integrity, we investigated the possible function of SOD1 in the proliferation and survival of ISC. We provided the first evidence that SOD1 promotes the ISC growth via suppressing WNT signaling and upregulating EREG expression, which is independent of SOD1 enzyme activity.

## Results

### SOD1 promotes organoid proliferation and survival

**Fig. 1:**
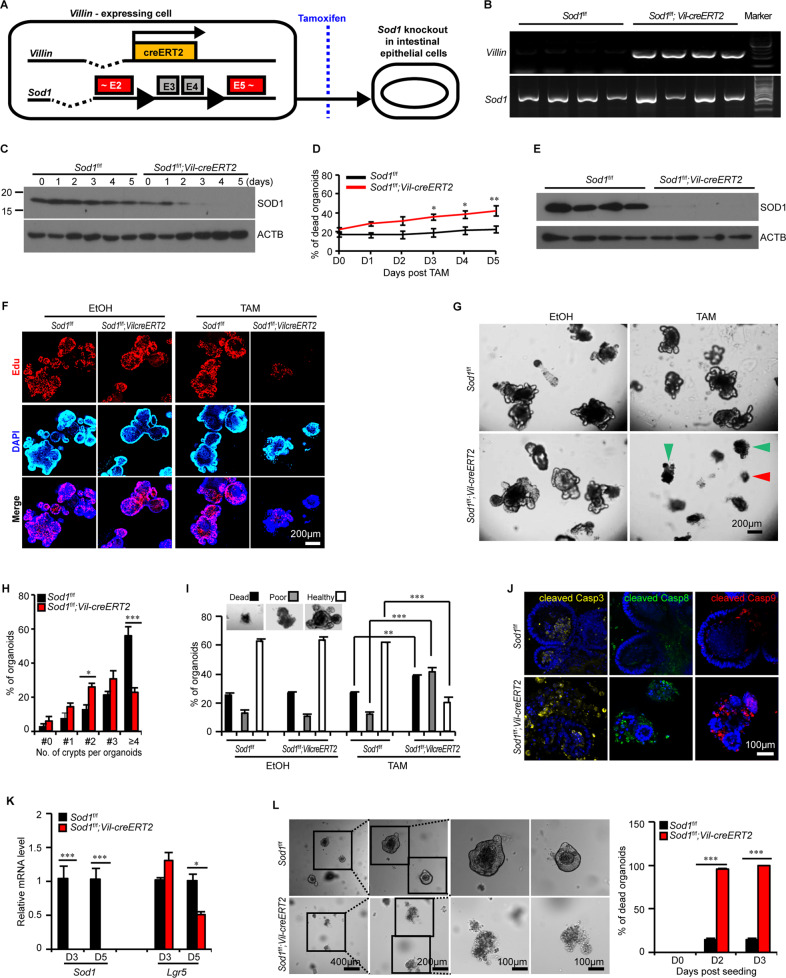
*Sod1* loss suppresses organoid proliferation and survival. **A** Experimental scheme for generation of *Sod1*^*f/f*^*; Vil-creERT2* mice. **B** Genotyping using PCR. **C** Western blot analysis of SOD1 protein in organoids from day 0 to day 5 after tamoxifen treatment. *Sod1*^*f/f*^ and *Sod1*^*f/f*^*; Vil-creERT2* organoids were treated with 2 μg ml^−1^ tamoxifen on day 0 (2 days after seeding) and analyzed for SOD1 expression from day 0 to day 5 (*n* = 4 mice per genotype). **D** Quantification of the percentage of dead organoids from day 0 to day 5 following tamoxifen treatment. TAM, tamoxifen. Data represent mean ± standard error of the mean (SEM), *n* = 6 wells per mouse; *n* = 4 mice per genotype. **E** Western blot analysis of SOD1 protein in organoids after 5 days of tamoxifen treatment (*n* = 4 mice per genotype). **F** Confocal images (nuclear staining, blue; and EdU staining, red) of Edu incorporation (1 h) in organoids after 5 days of tamoxifen or ethanol-only vehicle control treatment. Tamoxifen was dissolved in ethanol. EtOH, ethanol. **G** Brightfield images of organoids after 5 days of tamoxifen or ethanol-only vehicle controls treatment. Green arrowhead indicates “poor” organoids, and red arrowhead indicates dead organoids. **H** Percentage of organoids with 0, 1, 2, 3, or ≥4 crypts formed after 5 days of tamoxifen treatment. Data represent mean ± SEM; *n* = 6 wells per mouse; *n* = 4 mice per genotype; number of organoids counted: *n* = 504 (*Sod1*^*f/f*^), *n* = 557 (*Sod1*^*f/f*^*;Vil-creERT2*) organoids per group; one of three experiments. **I** Percentage of dead, poor, and healthy organoids analyzed after 5 days of tamoxifen or ethanol-only vehicle controls treatment. Data represent mean ± SEM; *n* = 6 wells per mouse; *n* = 4 mice per genotype; number of organoids counted: *n* = 511 (*Sod1*^*f/f*^ + ethanol), *n* = 656 (*Sod1*^*f/f*^*;Vil-creERT2* + ethanol), *n* = 498 (*Sod1*^*f/f*^ + tamoxifen), *n* = 784 (*Sod1*^*f/f*^*;Vil-creERT2* + tamoxifen) organoids per group; one of three experiments. **J** Fluorescence immunostaining to detect cleaved caspase-3 (yellow), cleaved caspase-8 (green) and cleaved caspase-9 (red) in the organoids after 5 days of tamoxifen treatment. Representative images of staining performed on organoids derived from 3 separate mice. **K** qPCR results showing relative mRNA expression of *Sod1* and *Lgr5* genes in organoids 3 and 5 days after tamoxifen treatment. Data represent mean ± SEM. *n* = 4 mice per genotype. **L** Organoids cultured for 2 days with *Sod1*-deficient crypts isolated from the *Sod1*^*f/f*^ and *Sod1*^*f/f*^*; Vil-creERT2* mice after 4 weeks post 5 consecutive tamoxifen injection (left); histogram showing quantification of the number of dead organoids on the indicated day after seeding (right). Data represent mean ± SEM. *n* = 3 mice per genotype. All results are representative of at least three independent experiments. All statistical significances were tested by two-way ANOVA. ^*^P < 0.05, ^**^P < 0.01, ^***^P < 0.001.

To elucidate SOD1 function in ISCs, we established 3D intestinal organoid cultures with freshly isolated crypts from tamoxifen–inducible intestinal epithelial cell (IEC)–specific *Sod1* knockout mice (*Sod1*^*f/f*^*; Vil-creERT2*) (Fig. 1A, B). After treating the cultures with tamoxifen, SOD1 expression in organoids was time-dependently inhibited and deleted completely on day 3 (Fig. 1C), accompanying by a time-dependent increase of the percentage of dead organoid, which was determined based on the absence of a defined edge, no light bud, overall dark appearance, and very small size (Fig. 1D). To evaluate the function of SOD1 in the organoid growth, we detected the proliferation and survival of organoids after 5 days of tamoxifen treatment. Western blot showed that SOD1 expression was completely blocked on day 5 (Fig. 1E). *Sod1*^*ΔIEC*^ organoids exhibited reduced Edu-positive cells (Fig. 1F) and decreased budding number per organoid (Fig. 1G, H), suggesting that *Sod1* loss inhibits the proliferation of organoids. Compared to control group which had higher percentage of “healthy” organoids which exhibited clean and light appearance, tightly packed cells and integral epithelia, *Sod1*-deficient group had a higher percentage of “dead” organoids and “poor” organoids which displayed more loosely packed cells, fewer light buds, overall dark appearance, and smaller size (Fig. 1G, I). Moreover, *Sod1* loss increased propidium iodide (PI)-positive cells in the epithelial layer of the organoids (Fig. S1A, B), and promoted fluorescent signals of active caspase-3, caspase-8 and caspase-9 in organoids (Fig. 1J), indicating that *Sod1* deletion inhibits the survival of organoids and promotes its apoptosis. qPCR analysis showed that the loss of *Sod1* significantly reduced the mRNA expression level of the ISC marker *Lgr5* (Fig. 1K). To further confirm the role of SOD1 in organoid growth, *Sod1*^*f/f*^ and *Sod1*^*f/f*^*; Vil-creERT2* mice were intraperitoneally injected with tamoxifen, and crypts were isolated for organoid culture after 4 weeks post 5 consecutive tamoxifen injection. *Sod1*-deficient crypt was cultured under standard conditions and almost completely dead 2 days after seeding (Fig. 1L). Thus, SOD1 is indispensable for organoid proliferation and survival.

### SOD1-mediated organoid growth is independent of its enzymatic activity

**Fig. 2:**
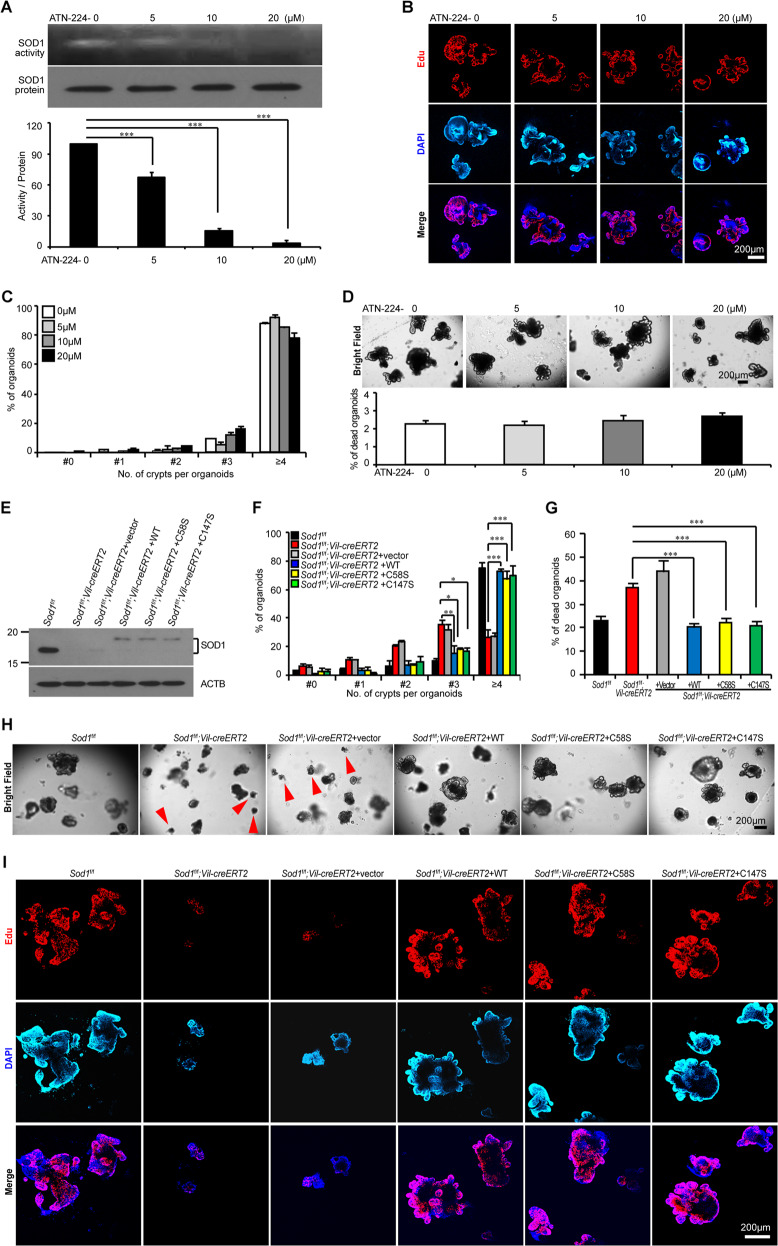
SOD1-mediated organoid growth is independent of its enzymatic activity. **A** *Sod1*^*f/f*^*; Vil-creERT2* organoids were treated with 0 (PBS), 5, 10, and 20 μM ATN-224. SOD1 protein level and enzymatic activity were analyzed after 5 days of ATN-224 treatment (upper panel). Histogram showing quantification of the brightness of SOD1 activity band (lower panel). Data represent mean ± SEM; *n* = 4 mice per genotype. Statistical significances were tested by one-way ANOVA. ^***^P < 0.001. **B** Confocal images (nuclear staining, blue; and EdU staining, red) of Edu incorporation in organoids after 5 days of ATN-224 treatment. **C** Percentage of organoids with 0, 1, 2, 3, or ≥4 crypts formed after 5 days of ATN-224 treatment. Data represent mean ± SEM; *n* = 6 wells per group; *n* = 4 mice per genotype; organoids number counted: *n* = 375 (0 μM), *n* = 287 (5 μM), *n* = 363 (10 μM), *n* = 544 (20 μM) organoids per group; one of three experiments. **D** Brightfield images of organoids after 5 days of ATN-224 treatment. (upper panel). Percentage of dead organoids analyzed after 5 days of ATN-224 treatment (lower panel). Data represent mean ± SEM; *n* = 6 wells per group, *n* = 4 mice per genotype; organoids number counted: *n* = 1299 (0 μM), *n* = 1090 (5 μM), *n* = 1182 (10 μM), *n* = 1235 (20 μM) organoids per group; one of three experiments. **E** The endogenous SOD1 expression in *Sod1*^*fl/fl*^ organoids and the doxycycline (Dox)-induced expression of wild-type SOD1, SOD1^C58S^, and SOD1^C147S^ in *Sod1*^*fl/fl*^*; Vil-creERT2* organoids were analyzed by Western blot after 5 days of tamoxifen and doxycycline induction. *n* = 3 mice per genotype. Vector, vector control; WT, wild-type *Sod1*; C58S, *Sod1*^C58S^; C147, *Sod1*^C147S^. **F** Percentage of organoids with 0, 1, 2, 3, or ≥4 crypts formed after 5 days of tamoxifen and doxycycline induction. Untransduced *Sod1*^*fl/fl*^ organoids are taken along as a reference. Data represent mean ± SEM; *n* = 6 wells per group; *n* = 3 mice per genotype. Statistical significances were tested by two-way ANOVA. ^*^P < 0.05, ^**^P < 0.01, ^***^P < 0.001. Organoids number counted: *n* = 86 (*Sod1*^*fl/fl*^), *n* = 157 (*Sod1*^*fl/fl*^*; Vil-creERT2*), *n* = 410 (*Sod1*^*fl/fl*^*; Vil-creERT2* + vector), *n* = 93 (*Sod1*^*fl/fl*^*; Vil-creERT2* + wild-type *Sod1*), *n* = 77 (*Sod1*^*fl/fl*^*; Vil-creERT2* + *Sod1*^C58S^), *n* = 81 (*Sod1*^*fl/fl*^*; Vil-creERT2* + *Sod1*^C147S^) organoids per group; one of three experiments. **G** Quantification of the percentage of dead organoids after 5 days of tamoxifen and doxycycline induction. Data represent mean ± SEM. *n* = 6 wells per group; *n* = 3 mice per genotyping. Statistical significances were tested by one-way ANOVA. ^***^P < 0.001. Number of organoids counted: *n* = 1008 (*Sod1*^*fl/fl*^), *n* = 1244 (*Sod1*^*fl/fl*^*; Vil-creERT2*), *n* = 760 (*Sod1*^*fl/fl*^*; Vil-creERT2* + vector), *n* = 1202 (*Sod1*^*fl/fl*^*; Vil-creERT2* + wild-type *Sod1*), *n* = 896 (*Sod1*^*fl/fl*^*; Vil-creERT2* + *Sod1*^C58S^), *n* = 978 (*Sod1*^*fl/fl*^*; Vil-creERT2* + *Sod1*^C147S^) organoids per group; one of three experiments. **H** Brightfield images of organoids after 5 days of tamoxifen and doxycycline induction. Red arrowheads indicate the dead organoids. **I** Confocal images (nuclear staining, blue; and EdU staining, red) of Edu incorporation in organoids after 5 days of tamoxifen and doxycycline induction. All results are representative of at least three independent experiments.

To determine the role of SOD1 enzymatic activity in SOD1-induced ISC growth, 0–20 μM of ATN-224, a specific inhibitor of SOD1 enzyme activity [34] was used. ATN-224 at 10 μM and 20 μM almost completely inhibited SOD1 activity in *Sod1*^*fl/fl*^*; Vil-creERT2* organoids (Fig. 2A). However, ANT-224 treatment did not affect organoid proliferation determined by Edu incorporation (Fig. 2B) and budding number per organoid (Fig. 2C), or organoid survival measured by PI staining (Fig. S2A, B) and the percentage of dead organoids (Fig. 2D).

Mutations in the catalytically active cysteine to serine (C58S or C147S) results in the loss of enzyme activity of SOD1 in yeast [35]. Both residues are conserved between mouse and yeast. To validate the functional significance of SOD1 enzymatic activity, we over-expressed *Sod1*^C58S^ and *Sod1*^C147S^_,_ which mimic the inactive state, and wild-type *Sod1* in the *Sod1*^*ΔIEC*^ organoids (Fig. 2E). *Sod1*^*fl/fl*^ organoids, expressing endogenous levels of *Sod1* was included as a reference. The results showed that inactive *Sod1* (*Sod1*^C58S^, *Sod1*^C147S^) and wild-type *Sod1* rescued the proliferation (Fig. 2F, I) and survival (Fig. 2G, H) of *Sod1*^*ΔIEC*^ organoids. All these data indicate that SOD1-induced organoid growth is independent of its enzymatic activity.

### Loss of *Sod1* does not affect the ROS levels on the organoid

**Fig. 3:**
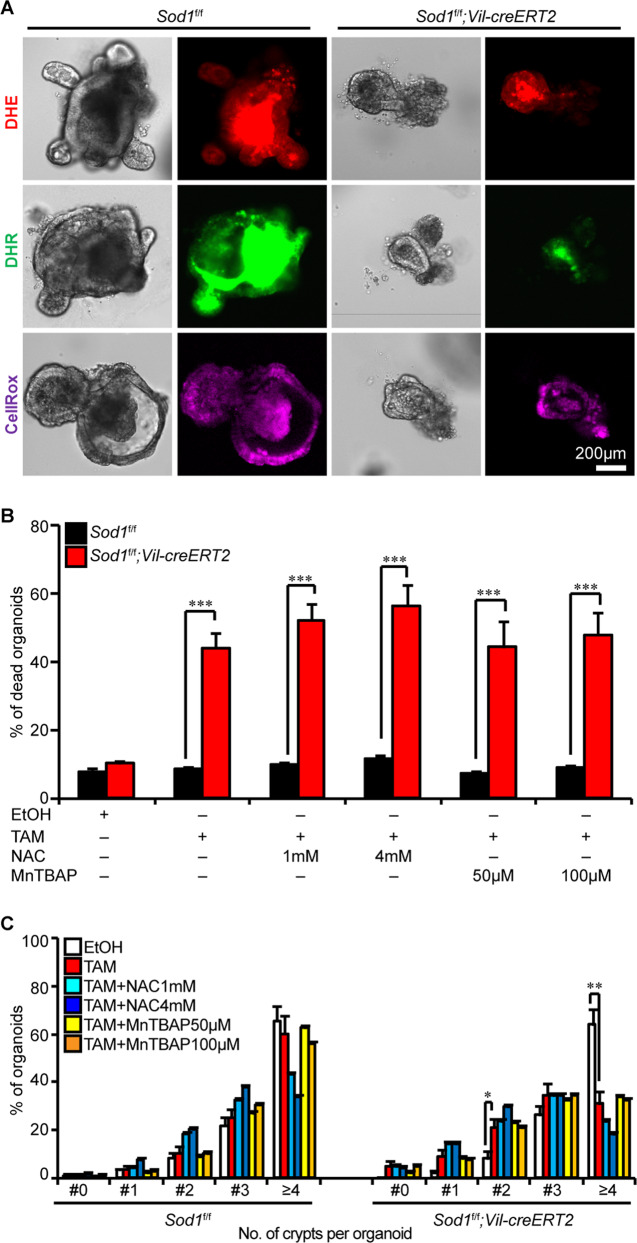
Loss of *Sod1* does not affect ROS levels in the organoid. **A** *Sod1* deletion does not affect superoxide level, peroxide level, and oxidative stress in the epithelial layer of organoids. *Sod1*^*f/f*^ and *Sod1*^*f/f*^*; Vil-creERT2* organoids were stained with DHE, DHR, and CellRox after 5 days of tamoxifen treatment. Representative images of staining performed on organoids. *n* = 4 mice per genotyping. **B** Quantification of the percentage of dead organoids after the indicated induction and antioxidant treatment. *Sod1*^*f/f*^ and *Sod1*^*f/f*^*; Vil-creERT2* organoid cultures were induced by ethanol or tamoxifen for 2 days and then treated with the indicated antioxidants (NAC at 1 mM and 4 mM, MnTBAP at 50 μM and 100 μM) for another 2 days. Data represent mean ± SEM. *n* = 6 wells per group; *n* = 4 mice per genotyping; organoids number counted: *n* = 1094 (*Sod1*^*f/f*^ + EtOH), *n* = 1070 (*Sod1*^*f/f*^ + TAM), *n* = 1107 (*Sod1*^*f/f*^ + TAM + NAC1mM), *n* = 1113 (*Sod1*^*f/f*^ + TAM + NAC4mM), *n* = 1150 (*Sod1*^*f/f*^ + TAM + MnTBAP50μM), *n* = 1202 (*Sod1*^*f/f*^ + TAM + MnTBAP100μM), *n* = 1316 (*Sod1*^*f/f*^*;Vil-creERT2* + EtOH), *n* = 841 (*Sod1*^*f/f*^*;Vil-creERT2* + TAM), *n* = 916 (*Sod1*^*f/f*^*;Vil-creERT2* + TAM + NAC1mM), *n* = 919 (*Sod1*^*f/f*^*;Vil-creERT2* + TAM + NAC4mM), *n* = 898 (*Sod1*^*f/f*^*;Vil-creERT2* + TAM + MnTBAP50μM), *n* = 936 (*Sod1*^*f/f*^*;Vil-creERT2* + TAM + MnTBAP100μM) organoids per group; one of four experiments. **C** Percentage of organoids with 0, 1, 2, 3, or ≥4 crypts formed after the indicated induction and antioxidant treatment. *Sod1*^*f/f*^ and *Sod1*^*f/f*^*; Vil-creERT2* organoid cultures were induced by ethanol or tamoxifen for 2 days and then treated with the indicated antioxidants (NAC at 1 mM and 4 mM, MnTBAP at 50 μM and 100 μM) for another 2 days. Data represent mean ± SEM. *n* = 6 wells per group; *n* = 4 mice per genotyping; organoids number counted: *n* = 421 (*Sod1*^*f/f*^ + EtOH), *n* = 391 (*Sod1*^*f/f*^ + TAM), *n* = 398 (*Sod1*^*f/f*^ + TAM + NAC1mM), *n* = 439 (*Sod1*^*f/f*^ + TAM + NAC4mM), *n* = 491 (*Sod1*^*f/f*^ + TAM + MnTBAP50μM), *n* = 472 (*Sod1*^*f/f*^ + TAM + MnTBAP100μM), *n* = 568 (*Sod1*^*f/f*^*;Vil-creERT2* + EtOH), *n* = 600 (*Sod1*^*f/f*^*;Vil-creERT2* + TAM), *n* = 547 (*Sod1*^*f/f*^*;Vil-creERT2* + TAM + NAC1mM), *n* = 521 (*Sod1*^*f/f*^*;Vil-creERT2* + TAM + NAC4mM), *n* = 634 (*Sod1*^*f/f*^*;Vil-creERT2* + TAM + MnTBAP50μM), *n* = 654 (*Sod1*^*f/f*^*;Vil-creERT2* + TAM + MnTBAP100 μM) organoids per group; one of four experiments. All data are representative of four independent experiments. All statistical significances were tested by two-way ANOVA. ^*^P < 0.05, ^**^P < 0.01, ^***^P < 0.001.

SOD1 plays a critical role in maintaining ROS homeostasis. ROS is known as a central regulator of ISC function [36]. Thus, we speculated that *Sod1* loss would cause elevated ROS, and thereby mediate growth defects in the organoids. However, *Sod1* loss did not alter the levels of superoxide and peroxide in the epithelial layer of organoids, as indicated by DHE staining for superoxide, DHR staining for peroxide and peroxynitrite, and CellROX staining for oxidative stress (Fig. 3A). To determine whether the growth defects in *Sod1*^*ΔIEC*^ organoid were due to ROS dysregulation, we treated the organoids with antioxidants such as the general ROS inhibitor NAC and the mitochondrial ROS inhibitor MnTBAP after two days of tamoxifen administration. As shown in Fig. 3B, C, the death rate and budding number in *Sod1*^*ΔIEC*^ organoids were not rescued after antioxidant treatment, even at higher concentrations. Organoids were then treated with tamoxifen and antioxidants simultaneously, which could not rescue the death rate of *Sod1*^*ΔIEC*^ organoids too (Fig. S3), indicating that ROS levels were not involved in *Sod1* loss-induced growth defects in the organoids.

### SOD1 promotes organoid growth by suppressing both WNT signaling and ectopic Paneth cell differentiation

**Fig. 4:**
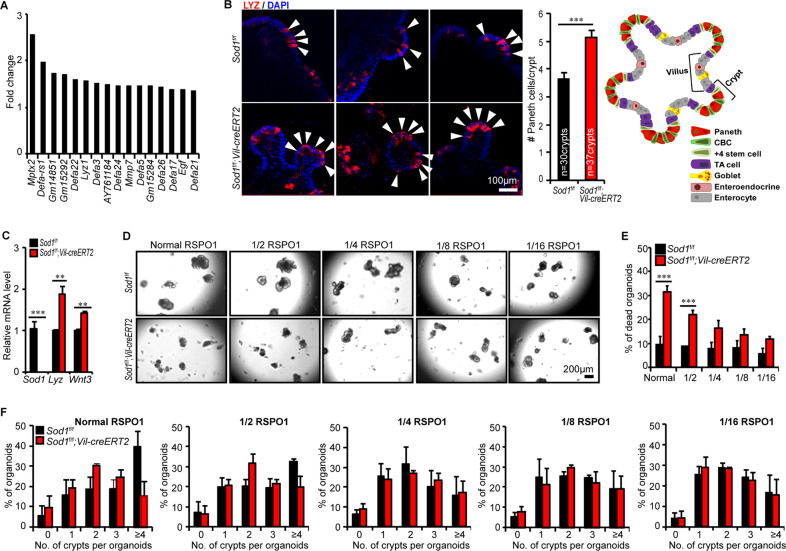
SOD1 supports organoid growth by suppressing WNT signaling and excessive Paneth cell differentiation. **A** Relative expression of Paneth cell signature genes by RNA-seq analysis are shown as a histogram comparing *Sod1*^*ΔIEC*^ and control organoids after 3 days of tamoxifen induction. *Sod1*^*f/f*^ and *Sod1*^*f/f*^*; Vil-creERT2* organoids were harvested after 3 days of tamoxifen induction for RNA-seq. *n* = 3 mice per genotype. **B** Fluorescence immunostaining to detect LYZ expression (red) in the organoids after 3 days of tamoxifen induction. Arrowheads indicate location of LYZ^+^ Paneth cells. Images are representative of stainings performed on organoids derived from 3 separate mice (left panel); Histogram (middle panel) shows the quantification of the number of LYZ^+^ Paneth cells per crypt, according to the scheme in the inset (right panel). The number of crypts measured per group was labeled on the histogram. One of three experiments. Data represent mean ± SEM; *n* = 6 wells per group; *n* = 3 mice per genotyping. Student *t*-test (two-tailed). ^***^P < 0.001. **C** qPCR analysis of *Sod1*, *Lyz*, and *Wnt3a* genes after 3 days of tamoxifen induction. Data represent mean±SEM; *n* = 3 mice per genotyping. Student *t*-test (two-tailed). ^**^P < 0.01, ^***^P < 0.001. **D** Representative images of *Sod1*^*f/f*^ and *Sod1*^*f/f*^*; Vil-creERT2* organoids treated with tamoxifen and cultured in reducing RSPO1 concentrations for 5 days. **E** Percentage of dead organoids assayed after 5 days in culture using indicated concentrations of RSPO1. Data represent mean±SEM; *n* = 6 wells per group; *n* = 3 mice per genotyping. Statistical significances were tested by two-way ANOVA. ^***^P < 0.001. Organoids number counted: *n* = 246 (*Sod1*^*f/f*^ + normal RSPO1), *n* = 385 (*Sod1*^*f/f*^*; Vil-creERT2* + normal RSPO1), *n* = 244 (*Sod1*^*f/f*^ + 1/2 RSPO1), *n* = 328 (*Sod1*^*f/f*^*; Vil-creERT2* + 1/2 RSPO1), *n* = 256 (*Sod1*^*f/f*^ + 1/4 RSPO1), *n* = 303 (*Sod1*^*f/f*^*; Vil-creERT2* + 1/4 RSPO1), *n* = 265 (*Sod1*^*f/f*^ + 1/8 RSPO1), *n* = 281 (*Sod1*^*f/f*^*; Vil-creERT2* + 1/8 RSPO1), *n* = 229 (*Sod1*^*f/f*^ + 1/16 RSPO1), *n* = 275 (*Sod1*^*f/f*^*; Vil-creERT2* + 1/16 RSPO1) organoids per group; one of three experiments. **F** Quantification of percentage of organoids with 0, 1, 2, 3, or ≥4 crypts formed after 5 days in culture using indicated concentrations of RSPO1. Data represent mean±SEM; *n* = 6 wells per group; *n* = 3 mice per genotyping; organoids number counted: *n* = 189 (*Sod1*^*f/f*^ + normal RSPO1), *n* = 203 (*Sod1*^*f/f*^*; Vil-creERT2* + normal RSPO1), *n* = 197 (*Sod1*^*f/f*^ + 1/2 RSPO1), *n* = 185 (*Sod1*^*f/f*^*; Vil-creERT2* + 1/2 RSPO1), *n* = 186 (*Sod1*^*f/f*^ + 1/4 RSPO1), *n* = 181 (*Sod1*^*f/f*^*; Vil-creERT2* + 1/4 RSPO1), *n* = 196 (*Sod1*^*f/f*^ + 1/8 RSPO1), *n* = 203 (*Sod1*^*f/f*^*; Vil-creERT2* + 1/8 RSPO1), *n* = 166 (*Sod1*^*f/f*^ + 1/16 RSPO1), *n* = 186 (*Sod1*^*f/f*^*; Vil-creERT2* + 1/16 RSPO1) organoids per group; one of three experiments. All results are representative of at least three independent experiments.

To elucidate the potential mechanism by which SOD1 promotes ISC growth, we performed RNA-seq using *Sod1*^*f/f*^ and *Sod1*^*f/f*^*;Vil-creERT2* organoids after 3 days of tamoxifen treatment. RNA-seq analysis showed that Paneth cell signature genes were 1.5−2.5 times higher in the *Sod1*-deficient organoids than in the controls (Fig. 4A). Consistently, the number of Paneth cells markedly increased in the *Sod1*^*ΔIEC*^ organoids (Fig. 4B). qPCR confirmed that the mRNA levels of the Paneth cell marker *Lyz* and *Wnt3* were significantly upregulated in *Sod1*^*ΔIEC*^ organoids (Fig. 4C). To determine whether elevated WNT signaling contributes to the organoid growth defects after *Sod1* knockout, we reduced WNT signaling in *Sod1*^*ΔIEC*^ organoids by reducing the levels of the WNT agonist RSPO1 [37]; this improved organoids survival despite reduced overall growth (Fig. 4D–F). These results indicate that, in the absence of *Sod1*, high WNT signaling-induced excessive Paneth cell differentiation plays a pivotal role in the appearance of growth defects in the organoids.

### Epithelial *Sod1* deficiency promotes apoptosis in the stem cell regions in vivo

**Fig. 5:**
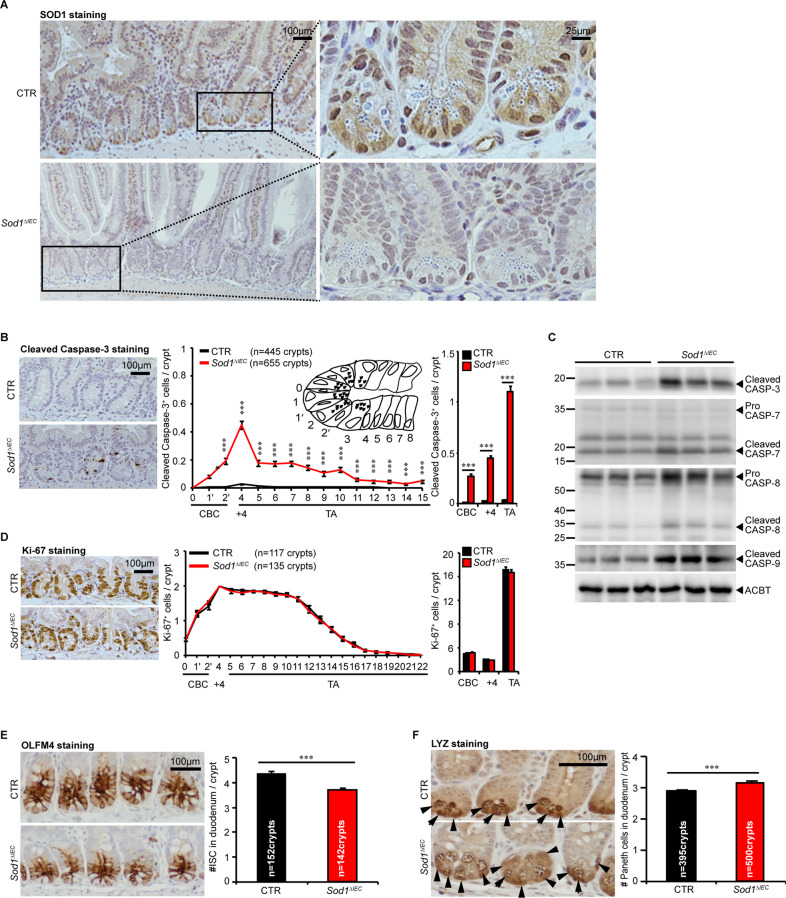
Epithelial *Sod1* loss promotes crypt ISC apoptosis and Paneth cell differentiation in vivo. **A** Representative IHC staining for SOD1 in small intestinal sections from *Sod1*^*f/f*^ and *Sod1*^*f/f*^*; Vil-creERT2* mice after 24 h post 5 consecutive tamoxifen injections. *n* = 4 mice per genotype. **B** Representative IHC staining for cleaved caspase-3 in small intestinal sections from *Sod1*^*f/f*^ and *Sod1*^*f/f*^*; Vil-creERT2* mice after 24 h post 5 consecutive tamoxifen injections (left panel); scoring of frequency of cleaved caspase-3-positive cells per crypt at specific positions relative to the crypt bottom, according to the scheme in the inset (middle panel). Statistical significances on the middle panel were tested by two-way ANOVA. Quantification of frequency of cleaved caspase-3-positive cells in three classes: CBC cells (located at position 0, 1′, 2′), +4 cells (located at position 4), and TA cells (located at position 5-15) (right panel). Statistical significance on the right panel was tested by Student *t*-test (two-tailed). ^***^P < 0.001. *n* = 4 mice per genotype; crypts number measured: *n* = 445 (CTR), *n* = 655 (*Sod1*^*∆IEC*^) crypts per group; one of three experiments. **C** Western blot for cleaved caspase-3, caspase-7, caspase-8, cleaved caspase-9 and Actin in the duodenal crypt samples of control and *Sod1*^*∆IEC*^ mice after 24 h post 5 consecutive tamoxifen injections. *n* = 3 mice per genotype. **D** Representative IHC staining for Ki-67 in small intestinal sections from *Sod1*^*f/f*^ and *Sod1*^*f/f*^*; Vil-creERT2* mice after 24 h post 5 consecutive tamoxifen injections (left panel); scoring of frequency of Ki-67-positive cells per crypt at specific positions relative to the crypt bottom (middle panel). Statistical significance on the middle panel were tested by two-way ANOVA. Quantification of frequency of Ki-67-positive cells in three classes: CBC cells (located at position 0, 1′, 2′), +4 cells (located at position 4), and TA cells (located at position 5-22) (right panel). Statistical significance on the right panel was tested by Student *t*-test (two-tailed). *n* = 4 mice per genotype; crypts number measured: *n* = 117 (CTR), *n* = 135 (*Sod1*^*∆IEC*^) crypts per group; one of three experiments. **E** Representative IHC staining for OLFM4 after 4 weeks post 5 consecutive tamoxifen injection (left panel); scoring of frequency of OLFM4-positive cells per crypt at specific positions (right panel). Statistical significance was tested by Student *t*-test (two-tailed). ^***^P < 0.001. *n* = 4 mice per genotype; number of crypts counted per group was labeled on the histogram. One of three experiments. **F** Representative IHC staining for LYZ in the small intestines of *Sod1*^*f/f*^ and *Sod1*^*f/f*^*; Vil-creERT2* mice after 4 weeks post 5 consecutive tamoxifen injection. Arrowheads point to the location of LYZ^+^ Paneth cells per crypt (left panel); right panel is the quantification of LYZ^+^ Paneth cells per crypt. Statistical significance was tested by Student *t*-test (two-tailed). ^***^P < 0.001. *n* = 4 mice per genotype; number of crypts measured per group is labeled on the histogram. One of three experiments. All results are representative of at least three independent experiments.

To determine whether SOD1 is important for ISCs in vivo, we first tested SOD1 localization. Immunohistochemistry revealed that in control mice, TA cells showed both nuclear and cytoplasmic SOD1 expression, and the crypt base cells including Paneth cells and CBCs exhibited stronger nuclear SOD1 expression (Fig. 5A). The villous epithelial cells showed only cytoplasmic SOD1 expression (data not shown). After 24 h post 5 consecutive days of tamoxifen injections, the SOD1 immunostaining in all epithelial cells was lost (Fig. 5A). SOD1 deletion in the epithelium significantly promoted the activation of caspase-3 in the stem cell regions (Fig. 5B). Consistently, western blot analyses using separated crypt tissues showed that *Sod1* loss induced higher expression of apoptotic proteins including cleaved caspase-3, cleaved caspase-7, cleaved caspase-8, and cleaved caspase-9 (Fig. 5C), suggesting that epithelial *Sod1* deficiency in vivo promotes apoptosis of crypt stem cells. However, *Sod1*^*ΔIEC*^ mice showed no significant changes in body weight, crypt-villus architecture, and cell proliferation in comparison to control mice (Fig. S4A, B and Fig. 5D). As depicted in Fig. 5E, F, *Sod1* deficiency in vivo also led to a lower number of ISCs expressing OLFM4, a marker of LGR5^+^ ISCs, and an increased Paneth cell numbers per crypt.

### EREG compensates for growth defects induced by *Sod1* deficiency

**Fig. 6:**
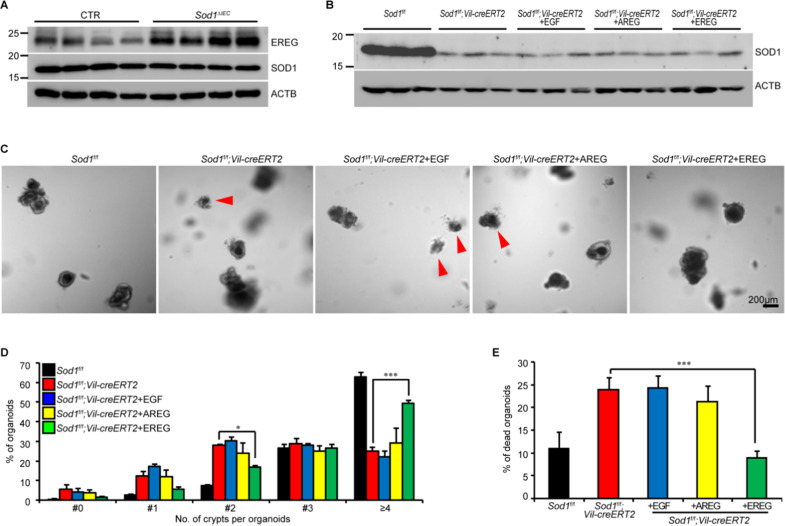
EREG rescues the growth defects in *Sod1* deficient organoids. **A** Western blot analysis of EREG and SOD1 expression using the separated stromal compartment from *Sod1*^*f/f*^ and *Sod1*^*f/f*^*; Vil-creERT2* mice after 24 h post 5 consecutive tamoxifen injections. *n* = 4 mice per genotype. **B** Western blot analysis of SOD1 expression in *Sod1*^*f/f*^ and *Sod1*^*f/f*^*; Vil-creERT2* organoids after 5 days of tamoxifen with or without 0.5 μg ml^-1^ of recombinant EGF/AREG/EREG induction. *n* = 3 mice per genotype. **C** Representative images of organoids after 5 days of tamoxifen with or without EGF/AREG/EREG induction. *n* = 6 wells per group; *n* = 3 mice per genotype. Red arrowhead indicates dead organoids. **D** Percentage of organoids with 0, 1, 2, 3, or ≥4 crypts formed after 5 days of tamoxifen with or without EGF/AREG/EREG induction. Data represent mean ± SEM; *n* = 6 wells per group; *n* = 3 mice per genotyping. Statistical significances were tested by two-way ANOVA. ^*^P < 0.05, ^***^P < 0.001. Number of organoids counted: *n* = 249 (*Sod1*^*f/f*^), *n* = 287 (*Sod1*^*f/f*^*; Vil-creERT2*), *n* = 307 (*Sod1*^*f/f*^*; Vil-creERT2* + EGF), *n* = 309 (*Sod1*^*f/f*^*; Vil-creERT2* + AREG), *n* = 272 (*Sod1*^*f/f*^*; Vil-creERT2* + EREG) organoids per group; one of three experiments. **E** Percentage of dead organoids assayed after 5 days of tamoxifen with or without EGF/AREG/EREG induction. Data represent mean ± SEM; *n* = 6 wells per group; *n* = 3 mice per genotyping. Statistical significance was tested by one-way ANOVA. ^***^P < 0.001. Number of organoids counted: *n* = 393 (*Sod1*^*f/f*^), *n* = 475 (*Sod1*^*f/f*^*; Vil-creERT2*), *n* = 537 (*Sod1*^*f/f*^*; Vil-creERT2* + EGF), *n* = 532 (*Sod1*^*f/f*^*; Vil-creERT2* + AREG), *n* = 413 (*Sod1*^*f/f*^*; Vil-creERT2* + EREG) organoids per group; one of three experiments. All results are representative of at least three independent experiments.

The EGFR ligand EREG is highly expressed in mesenchymal cells, and supports ISC growth [38–40]. We separated the stromal compartment from the small intestine of *Sod1*^*f/f*^ and *Sod1*^*ΔIEC*^ mice after 24 h post 5 consecutive tamoxifen injections. Western blot analysis showed enhanced EREG expression in the stromal tissue of *Sod1*^*ΔIEC*^ mice, and similar SOD1 expressions in the control and *Sod1*^*ΔIEC*^ stromal compartment which was because tamoxifen induced deletion of *Sod1* only in the epithelial cell lineages (Fig. 6A). Furthermore, treatment of *Sod1*^*ΔIEC*^ organoids showed that EREG rescued the morphology and growth of *Sod1*^*ΔIEC*^ organoids, and other EGFR ligands such as EGF and AREG did not (Fig. 6B, C, D, E). Thus, EREG could support the growth of *Sod1*^*ΔIEC*^ organoids, and stromal EREG upregulation might compensate for *Sod1* loss in vivo.

## Discussion

The mouse intestinal organoid is a useful ex vivo model for the study of ISCs, because it retains critical in vivo characteristics and normal numbers of ISCs and differentiated cell lineages [41]. ISCs, Paneth cells, and TA cells are localized at the bud site of organoids (crypt region). The crypt region undergoes continuous budding events surrounding a central lumen lined by a villus-like epithelium (villus region) [41]. The enterocyte, goblet cells and enteroendocrine cells are located at the villus region, and apoptotic cells are shed into the lumen in a manner similar to cell loss at the villus tips in vivo. Organoid cultures can be generated from dissociated crypts or single *Lgr5*^+^ cells in vitro, and passaged for more than 8 months without genetic alterations [41]. In this work, we used the organoid model to study the function of SOD1 in ISCs. Our study first demonstrates that SOD1 is critical for organoid growth and *Lgr5* gene expression. SOD1 is known for its canonical role as an antioxidant enzyme. Thus, we studied the role of SOD1 enzymatic activity in SOD1-induced ISC growth, and showed that the SOD1 inhibitor ATN-224, which almost completely blocks the enzymatic activity of SOD1, did not affect the proliferation and survival of organoid. Moreover, catalytically inactive-*Sod1* could reverse the growth defects induced by *Sod1* deficiency. These observations revealed that the well-known enzyme SOD1 has a novel function in ISCs which is independent of its enzymatic activity.

ROS signaling plays an important role in ISC function, as reviewed [36]. ROS levels in ISCs are low in comparison to those in differentiated cells in *Drosophila* [42]. Intracellular ROS levels must be tightly regulated; too much or too little ROS could inhibit cell growth [43]. Deletion of autophagy related 5 (*Atg5*)-mediated ROS production could reduce ISC count [44], whereas repression of *Nrf2*, a regulator of anti-oxidative responses, could promote proliferative ROS in *Drosophila* ISC lineages [42]. Although SOD1 is known as a ROS-scavenging enzyme, there have been conflicting reports on the impact of *Sod1* loss on intracellular ROS levels. *Sod1* loss in mice caused oxidative stress, which mediates multiple organ pathologies [45–48]. However, Wang et al. recently found that SOD1-induced reduction of tumor burden in KRAS-driven NSCLC mice model did not alter the ROS levels [28]. In this study, we also showed that *Sod1* deletion did not significantly affect ROS levels in the organoids, and treatment with general or mitochondrial antioxidants could not rescue the growth defects in *Sod1*-deficient organoids. These data indicate that ROS signaling is not involved in SOD1-induced organoid growth.

Paneth cell-derived WNT signaling is essential for ISC growth. Inhibition of WNT signaling or genetic removal of Paneth cells in vivo results in the loss of ISCs [15, 17, 18]. On the other hand, activation of WNT signaling or excessive Paneth cell differentiation also promotes apoptosis of the crypt region cells [20] or exhausts the ISC pool upon damage [40]. Here, we found notable increases in WNT signaling and Paneth cell number after *Sod1* deletion in organoids. This increase occurred before reduction of *Lgr5* gene expression (Fig. 1K and Fig. 4C). Furthermore, decreasing WNT signaling could block the growth defects in *Sod1*-deficient organoids. All these data indicate that redundant WNT signaling plays an important role in *Sod1* loss-induced organoid growth defect.

Epithelial *Sod1* deficiency in vivo inhibited the survival of the crypt stem cells but not the proliferation and crypt-villus architecture, while *Sod1* deficiency in organoids induced a significant inhibition of survival and proliferation. The *Sod1*-deficient crypt-derived organoids were almost completely dead 2 days after seeding (Fig. 1L). To explain this discrepancy, we studied the difference between intestinal organoids and the small intestine in vivo. Stromal/mesenchymal cells are absent in organoids [49], but present in vivo. These stromal cells provide essential niche factors for ISCs in vivo [50], and stromal EGFR signaling promotes ISC proliferation [40]. EGFR has seven known ligands: EGF, AREG, EREG, TGFA, HB-EGF, BTC, and EPGN [51]. Unlike the other ligands, EREG has more potent bioactivity [52, 53]. In this study, we also showed enhanced stromal EREG expression in *Sod1*^*ΔIEC*^ mice. Furthermore, supplementation of EREG, but not AREG and EGF, in vitro could rescue the growth of *Sod1* deficient organoids. These data indicate that elevated stromal EREG may compensate for *Sod1* loss in vivo, thereby preserving intestinal crypt-villus architecture.

In summary, our results showed that SOD1 promotes ISC growth independent of its enzyme activity, by suppressing both WNT signaling and excessive Paneth cell differentiation, while maintaining intestinal structure in vivo that may include EREG support. Paneth cells are frequently found in human colorectal adenomas and adenocarcinomas [54, 55]. Thus, our finding that SOD1 suppresses ectopic Paneth cell differentiation could suggest a self-sustaining mechanism that prevents intestinal tumor formation.

## Material and methods

### Mice

All mice were maintained in SPF housing, with a maximum of five mice per cage, under a 12 h light–dark cycle at a temperature range of 21 ± 2 °C. All mice were of C57/B6 and 129 mixed background. *Sod1*^*flox/flox*^ mice were obtained from Dr. Holly Van Remmen [56]. *Sod1*^*flox/flox*^ mice were bred with *Vil-creERT2* mice (JAX stock ♯ 020282) to generate *Sod1*^*flox/flox*^; *Vil-creERT2* mice. All mouse experiments were conducted in accordance with the National Institutes of Health Guidelines for the Care and Use of Laboratory Animals. The study procedures were approved by the Institutional Animal Care and Use Committee of Renji Hospital, Shanghai Jiao Tong University School of Medicine.

To genotype the animals, 2 mm of the ear or tail tissue was placed directly into 75 μl alkaline lysis buffer and heated in 95 °C 1 h. After heating, samples were cooled to 4 °C, treated with 75 μl neutralization buffer and analyzed by PCR. Experimental mice (8–16 weeks old) were injected intraperitoneally with 50 mg kg^−1^ tamoxifen (Sigma-Aldrich, Missouri, USA, T5648) in sunflower seed oil (Sigma-Aldrich, 88921) once a day for 5 consecutive injections. Both males and females were used. All mouse experiments were exclusively performed on littermate animals, with 3 to 5 mice used for each genotype. No randomization was done to determine how samples/ animals were allocated. All analyses of mouse phenotype were performed blinded.

### Organoid culture

Crypt isolation and culture were performed as previously described [57]. Briefly, duodenal crypts were obtained from *Sod1*^*flox/flox*^*; Vil-creERT2* and *Sod1*^*flox/flox*^ mice, seeded in growth factor reduced matrigel (BD, California, USA, 356231), and cultured in ENR (EGF, noggin, RSPO1) medium. Working ENR medium contained advanced DMEM/F12 (Thermo Scientific, Kansas, USA, 12634010), 1× Glutamax (Thermo Scientific, 35050061), 1× penicillin/streptomycin (Thermo Scientific, 15070063), 1× N2 Supplement (Thermo Scientific, 17502048), 1× B27 Supplement (Thermo Scientific, 12587010), 10 mol L^−1^ HEPES (Thermo Scientific, 15630-106), 1 mol L^−1^ N-acetyl l-cysteine (NAC) (Sigma-Aldrich, A9165), 0.05 μg ml^−1^ mouse recombinant EGF (Peprotech, New Jersey, USA, 315-09), 0.1 μg ml^−1^ mouse recombinant noggin (Peprotech, 250–38), and 5% RSPO1 (v/v). RSPO1 was produced using Cultrex HA-R-Spondin1-Fc 293 T (Trevigen, Maryland, USA). The organoids were passaged every 7 day by mechanically disrupting with a 20-Ga needle (Sigma-Aldrich, Z118052-100EA).

To stop SOD1 expression in the organoids, 2 μg ml^−1^ tamoxifen (Sigma-Aldrich, T5648) dissolved in ethanol was added to the culture medium on day 0 (2 days post seeding). The ethanol-only treatment is used as the vehicle control. The media was changed the next day, and subsequently every 2 days. The following inhibitors were used: ATN-224 (kindly provided by Dr. X.F.Steven Zheng, diluted in PBS), NAC (Sigma-Aldrich, A9165), and MnTBAP (Sigma-Aldrich, 475870). EGF (Peprotech, 315-09), Amphiregulin (AREG) (R&D Systems, Minnesota, USA, 989-AR-100), and EREG (R&D Systems, 1068-EP-050) were added to the culture media at a final concentration of 0.5 μg ml^−1^ on day 0 and were supplemented by media changes.

### Quantification of crypt number per organoid

Several random non-overlapping pictures were acquired from each well by using a Zeiss Axio Observer Z1 inverted microscope, and counted manually. Percentage of organoids with 0, 1, 2, 3, or ≥4 buddings were scored from 6 independent cultures for each mouse. There are 3-4 different mice per genotype. Organoids touching the edge of the images were excluded. The number of analyzed organoids is total number per group from one experiment.

### PI staining of organoids

PI staining was performed as described previously [58]. Briefly, the organoids were incubated with 500 nM propidium iodide (Sigma-Aldrich, P4170) in PBS solution at 37 °C for 20 min. The organoids were then washed with PBS and covered with new medium. Images were captured by Zeiss confocal microscope.

### Immunofluorescence

Organoids plated in 4-well chamber slides were fixed in 4% paraformaldehyde (PFA) for 30 min, permeabilized in 0.5% Triton for 20 min and blocked in 2% BSA. Mouse anti-lysozyme (Dako, Copenhagen, Denmark, A009902-2, 1:10000) primary antibody, cleaved caspase-3 (Cell Signaling Technology, Massachusetts, USA, 9664, 1:500) primary antibody, cleaved caspase-8 (Cell Signaling Technology, 8592, 1:500) primary antibody, cleaved caspase-9 (Cell Signaling Technology, 9507, 1:500) primary antibody and donkey anti-rabbit (Thermo Scientific, 1:2000) secondary antibody were used for immunostaining. Organoids were mounted with antifade mounting medium with DAPI (SouthernBiotech, Alabama, USA, 0100-20) for visualization. For EdU incorporation experiments, the intact organoid cultures were subjected to 1 h pre-incubation with EdU in ENR medium before fixing. Cells were stained using a Click-it EDU Imaging kit (Thermo Scientific, C10338), according to the manufacturer’s instructions. Images were acquired using a Zeiss confocal microscope.

### Detection of intracellular ROS, superoxide levels, and oxidative stress

Dihydroethidium (DHE) (Thermo Scientific, D23107), Dihydrorhodamine (DHR) (Thermo Scientific, D23806), and CellROX (Thermo Scientific, C10422) staining were performed using kits according to the manufacturer’s instructions. Briefly, ROS was monitored by staining the organoids with 10 μM DHR for 15 min at 37 °C. Organoids were then washed with PBS and covered with new ENR medium. To monitor intracellular superoxide levels, organoids were incubated with 1 μM DHE for 10 min at 37 °C. Organoids were then washed with PBS and covered with new ENR medium. To measure intracellular oxidative stress, the organoids were treated with 5 μM CellROX for 30 min at 37 °C. Organoids were then washed with PBS and covered with new ENR medium. Images were obtained using a Zeiss confocal microscope.

### Crypt and organoid protein extraction and western blotting

Duodenal crypts were insolated according to O’Rourke et al. [59]. Organoids were washed once with cold PBS and collected in cell recovery solution (Corning, New York, USA, 354253) to remove the matrigel. Duodenal crypts or organoid pellets were homogenized in cold RIPA lysis buffer (Beyotime Biotechnology, China, P0013C) containing proteinase and phosphatase inhibitors (Beyotime Biotechnology) for 10–20 min, sonicated at 4 °C using a Bioruptor® Plus device, mixed with 6×SDS loading buffer and heated at 100 °C for 5 min. Protein concentrations were measured using a Pierce™ BCA Protein Assay Kit (Thermo Scientific, 23225). Western blot analysis was performed using standard techniques [60]. The primary antibodies used were SOD1 (Santa Cruz Biotechnology, Texas, USA, sc-11407, 1:2000), epiregulin (Santa Cruz Biotechnology, sc-376284, 1:500), cleaved caspase-3 (Cell Signaling Technology, 9664, 1:1000), caspase-7 (Cell Signaling Technology, 9492, 1:1000), caspase-8 (Cell Signaling Technology, 4790, 1:1000), cleaved caspase-9 (Cell Signaling Technology, 9507, 1:1000) and β-Actin (Cell Signaling Technology, 4970, 1:1000).

### RNA extraction and qPCR

RNA from the organoids was extracted using the RNeasy Kit (Qiagen, Limburg, Netherlands, 74104) with on-column DNase treatment. cDNA was synthesized using a RETROscript^®^ Reverse Transcription Kit (Thermo Scientific, AM1710). qPCR was performed using PowerUp^™^ SyGreen Mix (Thermo Scientific, A25741). Normalization was done using the housekeeping gene *Hmbs*. *Sod1*-F: 5′-GAGGGTAGCAGATGAGTCTGAG-3′, *Sod1*-R: 5′-GAGTCTTGTTGCTAAGTAGAG-3′;*Lgr5*-F:5′-CCTACTCGAAGACTTACCCAGT-3′, *Lgr5*-R: 5′-GCATTGGGGTGAATGATAGCA-3′;*Lyz*-F:5′-ACTCCTCCTTGCTTTCTGTC-3′, *Lyz*-R: 5′-GTCGGTGCTTCGGTCTC-3′; *Wnt3*-F: 5′-TGGAACTGTACCACCATAGATGAC-3′, *Wnt3*-R: 5′-ACACCAGCCGAGGCGATG-3′; *Hmbs*-F:5′-GATGGGCAACTGTACCTGACTG-3′, *Hmbs*-R: 5′-CTGGGCTCCTCTTGGAATG-3′.

### Lentiviral transduction in organoids

Mouse wild-type *Sod1*, *Sod1*^C58S^, and *Sod1*^C147S^ (kindly provided by Dr. X.F.Steven Zheng) plasmids were cloned into the tet-inducible lentiviral vector pINDUCER20 (Addgene, Massachusetts, USA, 44012). For virus production, the *Sod1* plasmids were co-transfected with psPAX2 (Addgene, 12260), and pMD2.G (Addgene, 12259) into HEK293T cells using FuGENE® 6 Transfection Reagent (Promega, Wisconsin, USA, E2691). Viral supernatants were collected 24 and 48 h after transfection by passage through a 0.45 mm filter, and concentrated with lentivirus concentration solution (OriGene Technologies, Maryland, USA, TR30025) according to the manufacturer’s instructions.

Organoids were dissociated into single cells and transfected with concentrated lentiviruses as described previously [61]. Briefly, concentrated viral supernatants were added to cells in 48-well plates, incubated at 37 °C for 3 h, collected, and reseeded in Matrigel-containing medium. Infected organoids were selected with G418 (Thermo Scientific, 10131035) at 72 h after viral transduction for 2 weeks.

To induce exogenous vector-, wild-type *Sod1*, *Sod1*^C58S^, and *Sod1*^C147S^ expression in *Sod1*-deficient organoids, 1 μg ml^−1^ doxycycline (Sigma-Aldrich, D9891) was added to the culture media on day 0 and supplemented with media changes at 2-day intervals.

### Immunohistochemistry

Dissected intestines were fixed overnight in 4% paraformaldehyde and subsequently embedded in paraffin. Immunohistochemistry (IHC) was carried out according to standard procedures. The following primary antibodies were used for immunostaining: SOD1 (Abcam, Massachusetts, USA, ab16831, 1:500), cleaved caspase-3 (Cell Signaling Technology, 9664, 1:500), Ki-67 (Cell Signaling Technology, 12202, 1:500), OLFM4 (Cell Signaling Technology, 39141, 1:500), lysozyme (Dako, A009902-2, 1:10000) and goat anti-rabbit IgG secondary antibody (Vector Laboratories, DI-1594-1.5, 1:5000). VECTASTAIN Elite ABC HRP Kit (Vector Laboratories, California, USA, PK-6100) and ImmPACT DAB Peroxidase (HRP) Substrate (Vector Laboratories, SK-4105) were used to detect primary antibodies for Immunohistochemistry.

### SOD1 activity assay

SOD1 activity assay was carried out as previously described [62]. Briefly, organoids were harvested in cold PBS, lysed by sonication in phosphate buffer (0.05 M KH2PO4 and K2HPO4, pH 7.8) supplemented with 0.1% Triton X-100, and protease and phosphatase inhibitor cocktails. Organoid proteins (150–200 μg per sample) were loaded in 12% native gel. The native PAGE gels were stained with 2.43 mM nitro blue tetrazolium chloride (Sigma-Aldrich, N6876), 0.14 M riboflavin-5′-phosphate (Sigma-Aldrich, F1392), and 28 mM TEMED (Bio-Rad Laboratories, California, USA, 1610800) for 20 min at room temperature in darkness. To visualize SOD1 activity, gels were rinsed with water twice and placed on a light box for 15–120 min.

### RNA sequencing

Whole crypts were isolated from 9-weeks-old mice for organoid cultures, with three mice per genotype (*Sod1*^*f/f*^ and *Sod1*^*f/f*^*;Vil-creERT2*). Total RNA was isolated from organoids after 3 days of tamoxifen treatment using Rneasy mini plus kit (Qiagen). The concentration and quality of total RNA samples was first assessed using Agilent 2100 Bioanalyzer. A RIN (RNA Integrity Number) of five or higher was required to pass the quality control. Then four hundred nanograms of RNA per sample were used to prepared dual-indexed strand-specific cDNA library using TruSeq RNA Access Library Prep Kit (Illumina). Samples were processed on an Illumina HiSeq 4000 for 2 × 75 bp paired-end sequencing. Analysis of RNA-Seq data was carried out using the following protocol: the sequencing data were first assessed using FastQC (Babraham Bioinformatics, Cambridge, UK) for quality control. Then all sequenced libraries were mapped to the mouse genome (UCSC mm10) using STAR RNA-seq aligner [63]. The reads distribution across the genome was assessed using bamutils (from ngsutils) [64]. Uniquely mapped sequencing reads were assigned to mm10 refGene genes using featureCounts (from subread) [65]. Quality control of sequencing and mapping results was summarized using MultiQC [66]. Genes with read count per million (CPM) > 0.5 in more than 3 of the samples were kept. The data was normalized using TMM (trimmed mean of M values) method. Differential expression analysis was performed using edgeR [67, 68]. False discovery rate (FDR) was computed from *p*-values using the Benjamini-Hochberg procedure. All expression data were deposited in Gene Expression Omnibus (GEO accession number GSE211035).

### Statistical analysis

All data were presented as mean ± SEM and were analyzed using the GraphPad Prism 9 software. Statistical analyses were performed with two-way ANOVA, one-way ANOVA or Student’s *t* test. ^*^P < 0.05 was considered to be statistically significant.

## Supplementary information

Checklist

Supplementary Figure 1

Supplementary Figure 2

Supplementary Figure 3

Supplementary Figure 4

Supplementary Figure 5

Legends for Supplementary Figures

## Data Availability

The authors declare that all the other data supporting the findings of this study are available within the article and its Supplementary Information files and from the corresponding author on request.
