# Supplementary figures and images for: Non-enzymatic role of SOD1 in intestinal stem cell growth
Source: Cell Death Dis. 2022 Oct 20;13(10):882. doi: 10.1038/s41419-022-05267-w (PMC9585064; doi:10.1038/s41419-022-05267-w)

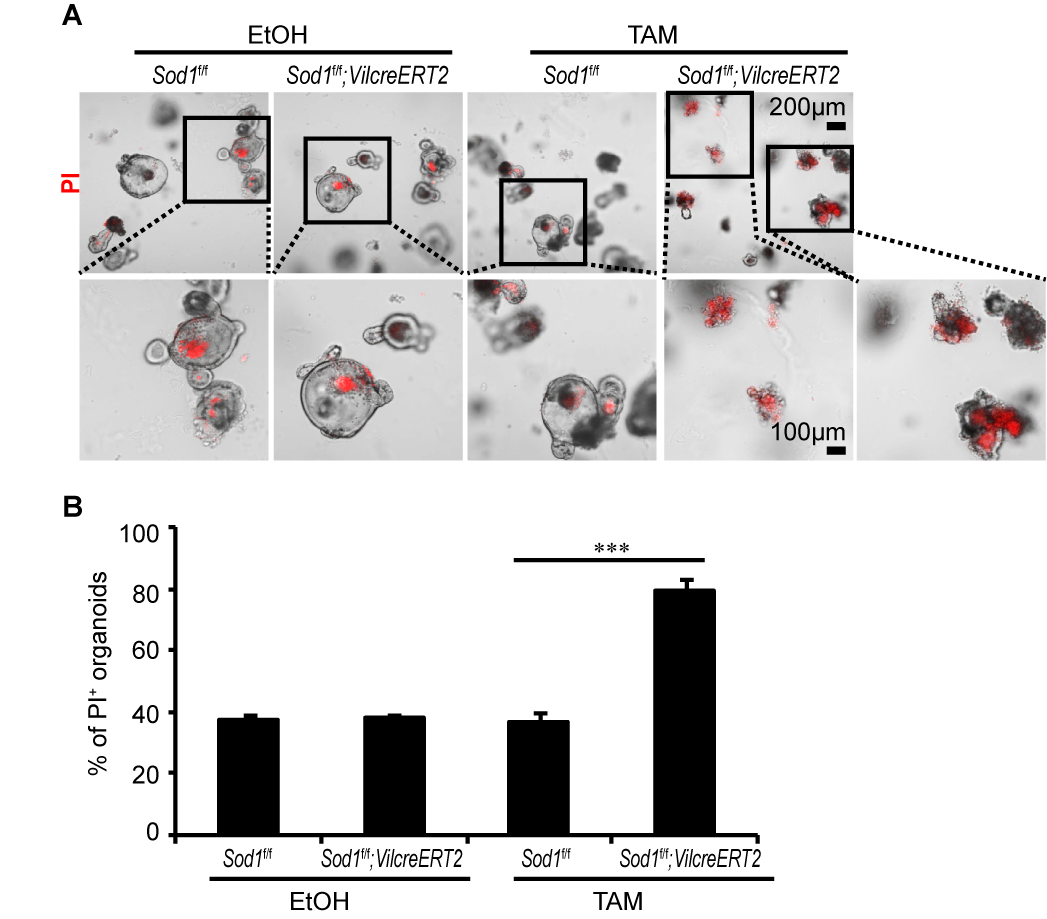

Supplement: Supplementary file 2 — Supplementary Figure 1 [file 41419_2022_5267_MOESM2_ESM.tif]

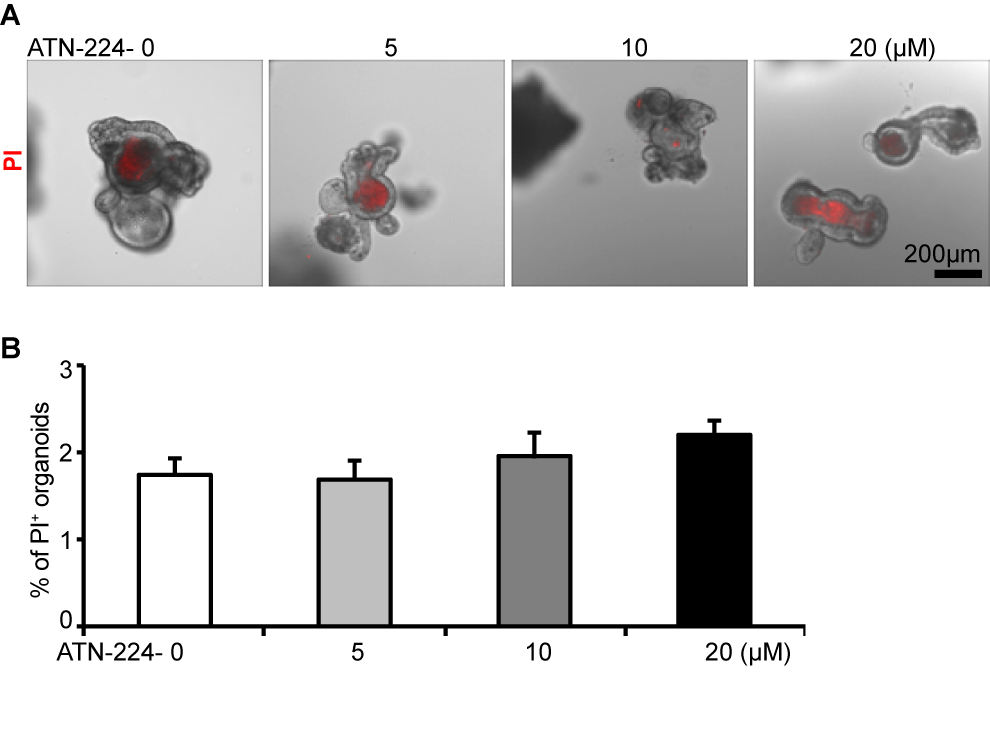

Supplement: Supplementary file 3 — Supplementary Figure 2 [file 41419_2022_5267_MOESM3_ESM.tif]

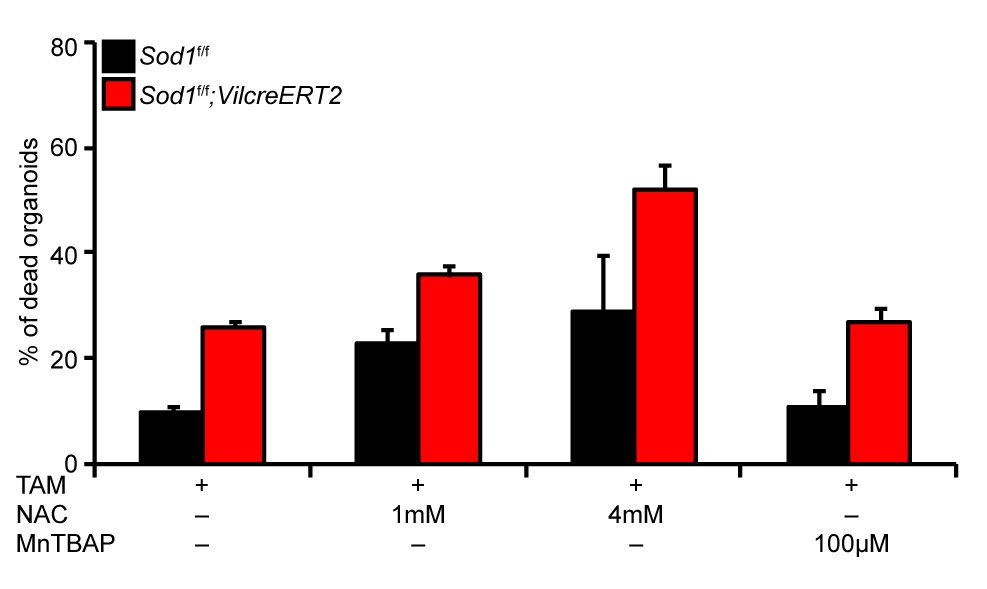

Supplement: Supplementary file 4 — Supplementary Figure 3 [file 41419_2022_5267_MOESM4_ESM.tif]

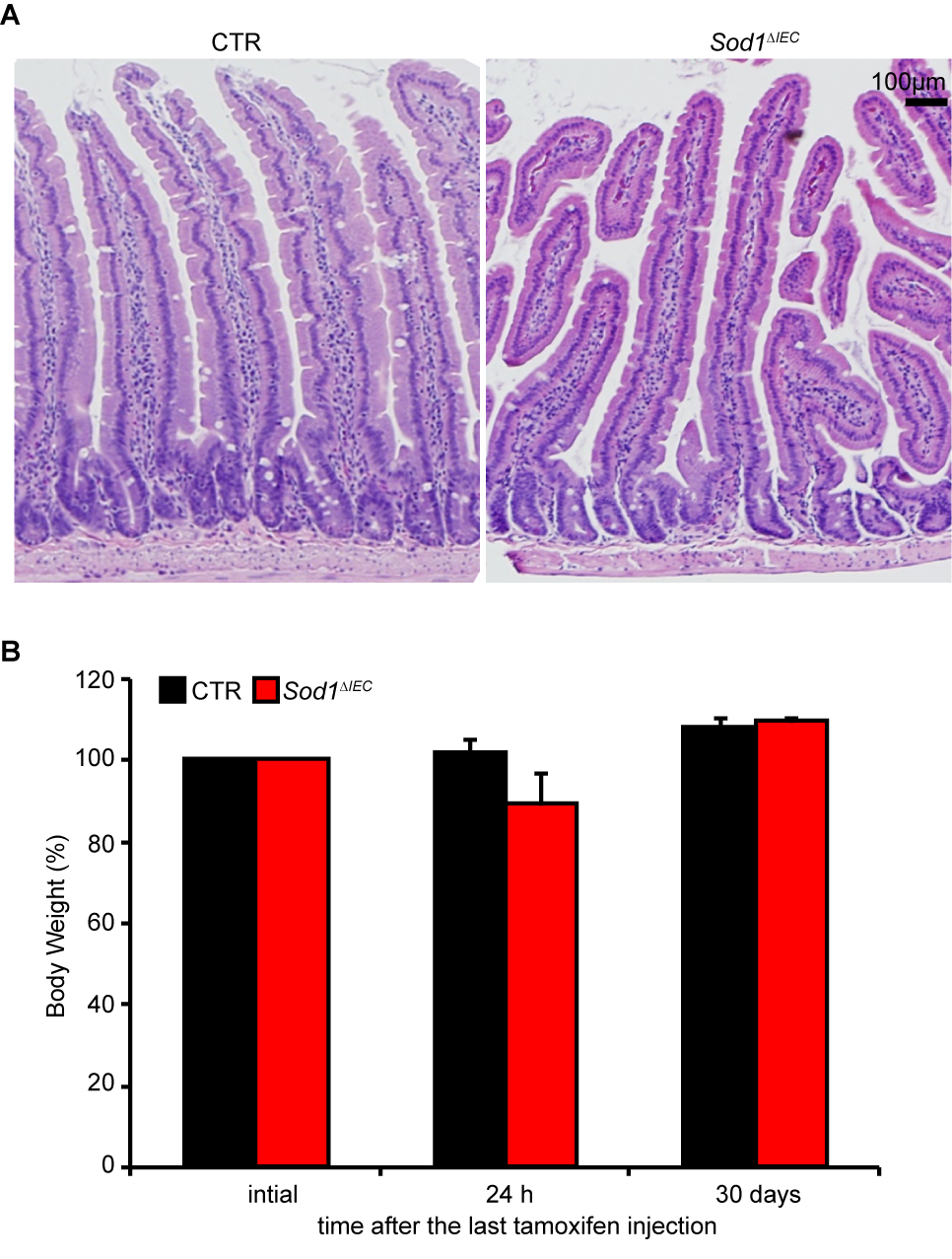

Supplement: Supplementary file 5 — Supplementary Figure 4 [file 41419_2022_5267_MOESM5_ESM.tif]

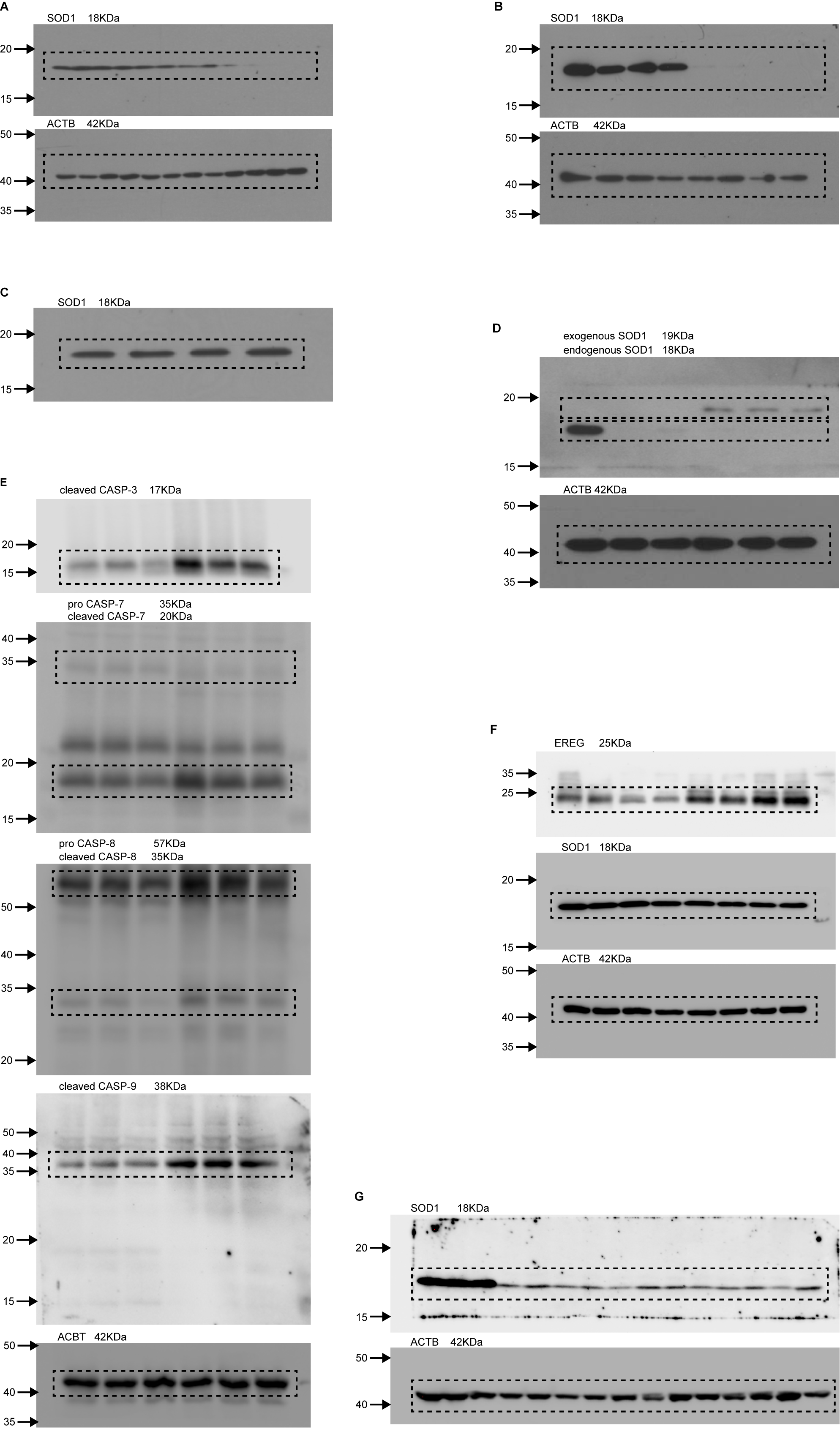

Supplement: Supplementary file 6 — Supplementary Figure 5 [file 41419_2022_5267_MOESM6_ESM.tif]
