# Supplementary material for: Non-enzymatic role of SOD1 in intestinal stem cell growth
Source: Cell Death Dis. 2022 Oct 20;13(10):882. doi: 10.1038/s41419-022-05267-w (PMC9585064; doi:10.1038/s41419-022-05267-w)
Supplement: Supplementary file 7 — Legends for Supplementary Figures [file 41419_2022_5267_MOESM7_ESM.docx]

**Legends for Supplementary Figures**

**Fig. S1 *Sod1* loss increases the number of organoid with PI-positive cells in the epithelial layer.** **A** *Sod1^f/f^* and *Sod1^f/f^;Vil-creERT2* organoids were stained with PI (red) to identify dead cells in the epithelial layer after 5 days of ethonal or tamoxifen induction. **B** Quantification of the percentage of organoids with PI-positive cells in the epithelial layer. Organoids viability was observed by confocal microscopy. Data represent mean ± SEM; n = 6 wells per mouse; n = 4 mice per genotype. All data are representative of three independent experiments. Statistical significances were tested by one-way ANOVA. ^***^*P*<0.001.

**Fig. S2 Inhibition of SOD1 activity does not affect the number of organoid with PI-positive cells in the epithelial layer.** **A** *Sod1^f/f^; Vil-creERT2* organoids were stained by PI (red) to identify dead cells in the epithelial layer after 5 days of 0, 5, 10, 20 μM ATN-224 treatment. **B** Quantification of the percentage of organoids with PI-positive staining in the epithelial layer. Organoids viability was observed by confocal microscopy. Data represent mean ± SEM; n = 6 wells per mouse; n = 4 mice per genotype. All data are representative of three independent experiments. Statistical significances were tested by one-way ANOVA.

**Fig. S3 Antioxidants do not rescue the survival of *Sod1*-deficient organoids.** Quantification of the percentage of dead organoid after the treatment of tamoxifen and antioxidants. *Sod1^f/f^* and *Sod1^f/f^; Vil-creERT2* organoid cultures were induced by tamoxifen and indicated antioxidants (NAC at 1mM and 4mM and MnTBAP at 100μM) simultaneously for 5 days. Data represent mean ± SEM; n = 6 wells per mouse; n = 4 mice per genotype; organoids number counted: n = 460 (*Sod1^f/f^*+TAM), n = 223 (*Sod1^f/f^;Vil-creERT2*+TAM), n = 427 (*Sod1^f/f^*+TAM+NAC1mM), n = 229 (*Sod1^f/f^;Vil-creERT2*+TAM+NAC1mM), n = 444 (*Sod1^f/f^*+TAM+NAC4mM) , n = 246 (*Sod1^f/f^;Vil-creERT2*+TAM+NAC4mM), n = 323 (*Sod1^f/f^*+TAM+MnTBAP100μM), n = 220 (*Sod1^f/f^;Vil-creERT2*+TAM+MnTBAP100μM) organoids per group; one of three experiments. All data are representative of three independent experiments. Statistical significances were tested by two-way ANOVA.

**Fig. S4 *Sod1* deletion in the intestinal epithelium does not affect the crypt-villus architecture and mice body weight. A** H&E staining of *Sod1^f/f^* and *Sod1^f/f^; Vil-creERT2* mice after 24 h post 5 consecutive days of tamoxifen injection. n=4 mice per genotype. **B** Body weight was measured at the indicated time. Initial, initial weight before the first tamoxifen injection; 24 h, the body weight after 24 h post the last tamoxifen injection; 30 days, the body weight after 30 days post the last tamoxifen injection. Normalized body weight was expressed as a percentage of the initial weight. Data represent mean ± SEM; n = 4 mice per genotype. All data are representative of four independent experiments. Statistical significance was tested by two-way ANOVA.

**Fig. S5 Uncropped immunoblots of the different figures. A** The original western blots of Figure 1C, immunoblots of SOD1 with β-actin obtained from small intestinal organoid lysate. **B** The original western blots of Figure 1E, immunoblots of SOD1 with β-actin obtained from small intestinal organoid lysate. **C** The original western blots of Figure 2A, immunoblots of SOD1 obtained from small intestinal organoid lysate. **D** The original western blots of Figure 2E, immunoblots of SOD1 with β-actin obtained from small intestinal organoid lysate. **E** The original western blots of Figure 5C, immunoblots of cleaved caspase-3, pro caspase-7, cleaved caspase-7, pro caspase-8, cleaved caspase-8, cleaved caspase-9, with β-actin obtained from small intestinal crypt tissue lysate. **F** The original western blots of Figure 6A, immunoblots of EREG, SOD1, with β-actin obtained from stromal tissue lysate. **G** The original western blots of Figure 6B, immunoblots of SOD1 with β-actin obtained from small intestinal organoid lysate.
